# IFN-γ-mediated suppression of ANGPT2-Tie2 in endothelial cells facilitates tumor vascular normalization during immunotherapy

**DOI:** 10.3389/fimmu.2025.1551322

**Published:** 2025-04-30

**Authors:** Zihao Cai, Kelin Meng, Taiyan Yu, Yu Xi, Zhiwei Yuan, Xue Wang, Congjian Wang, Lequn Li, Xiangning Fu

**Affiliations:** Thoracic Surgery Laboratory, Department of Thoracic Surgery, Tongji Hospital, Tongji Medical College, Huazhong University of Science and Technology, Hubei, Wuhan, China

**Keywords:** angiogenesis, ANGPT2, anti-PD-L1 therapy, IFN-γ, Tie2

## Abstract

**Introduction:**

Tumor angiogenesis is a critical biological hallmark of cancer, which involves multiple molecularly regulated signaling pathways, including the angiopoietin (ANGPT)-Tie2 and the vascular endothelial growth factor (VEGF) signaling pathways. Despite initial optimism, targeting tumor angiogenesis in the treatment of lung adenocarcinoma (LUAD) has been unsatisfactory. Currently, monotherapy with PD-1/PD-L1 inhibitors, or their combination with bevacizumab, is considered the standard therapeutic approach for LUAD. Recent studies have shown that immunotherapy suppresses tumor angiogenesis and facilitates vascular normalization. However, whether and how anti-PD-L1 therapy influences tumor vasculature remains unclear.

**Methods:**

To investigate the impact of immunotherapy on the vasculature of LUAD, a mouse model of lung adenocarcinoma was established by subcutaneous implantation of Lewis lung carcinoma cells *in vivo*. The effects of different treatments on microvessel density and pericyte coverage were explored, and the expression of angiogenesis-related factors was analyzed. Furthermore, to explore the molecular mechanisms through which IFN-γ regulates tumor blood vessels during immunotherapy, we elucidated the specific mechanisms *in vitro* by means of techniques such as siRNA, ChIP, RT-qPCR, Western blot, and immunofluorescence. Finally, the effects of IFN-γ on the proliferation, migration, and angiogenic function of endothelial cells (ECs) were evaluated through CCK-8, Transwell, and HUVEC tube formation assays.

**Results:**

Employing a mouse model of LUAD, we demonstrated that PD-L1 blockade therapy inhibits tumor angiogenesis and normalizes vasculature in an IFN-γ-signaling-dependent manner. Notably, anti-PD-L1 therapy reduced Tie2 and ANGPT2 expression, and these effects were reversed by the JAK1/2 inhibitor. Mechanistically, we demonstrated that IFN-γ inhibited Tie2 and ANGPT2 expression in ECs, and suppressed *ANGPT2* gene transcription through the AKT-FOXO1 signaling pathway. Interestingly, IFN-γ-mediated activation of STAT1 exerts negative regulation by directly binding to the promoter regions of the *ANGPT2* and *TEK* genes. Functionally, IFN-γ limits the migration, proliferation, and tube formation of ECs.

**Discussion:**

In conclusion, our results revealed a novel mechanism wherein IFN-γ-mediated inhibition of ANGPT2-Tie2 facilitates vascular normalization during immunotherapy in LUAD, which performs an essential function in the antitumor efficacy of immunotherapy.

## 1 Introduction

Lung cancer has long been the primary malignant tumor worldwide (1). Tumor angiogenesis in lung adenocarcinoma (LUAD), the most prevalent type of lung cancer, is widely acknowledged to play a pivotal role in tumor progression and immune evasion (2). Tumor angiogenesis is a multifaceted process characterized by the intricate interactions among various cell types, with endothelial cells (ECs) playing the most critical role (3, 4). The functions of ECs include proliferation, migration, and tube formation, which are modulated via complicated signal transduction pathways (5). The angiopoietin (ANGPT)-Tie2 signaling pathway plays a vital role in both angiogenesis and the remodeling and maturation of vascular structures (6, 7). ANGPT1 and ANGPT2, in conjunction with their receptor Tie2, are integral for the maturation and modification processes following angiogenesis. Specifically, ANGPT1 interacts with Tie2 to facilitate vascular stabilization, whereas ANGPT2 engages with Tie2 to induce vascular destabilization (8, 9). Therefore, targeting ANGPT2 can promote vascular normalization and improve the tumor microenvironment (10–12).

Over recent years, immune checkpoint inhibitors (ICIs) have emerged as pivotal modalities in cancer therapy. Nevertheless, the use of ICIs is limited. Hence, for clinical application, researchers have proposed integrating antiangiogenic therapy to mitigate the limitations of ICIs (13, 14). However, the effectiveness of this treatment remains suboptimal (15, 16), which has led researchers to investigate the interaction between ICIs and the tumor vasculature (17). The combination blockade of programmed death-protein 1 (PD-1) and cytotoxic T-lymphocyte antigen 4 promotes vascular normalization upon the activation of CD4^+^ T lymphocytes (18). Furthermore, anti-PD-1/programmed death-ligand 1 (PD-L1) antibodies regulate angiogenesis by CD8^+^ T cells or tumor-derived CXCL10/11 (19, 20). Moreover, anti-PD-L1 therapy can suppress the nuclear expression of PD-L1 in cancer cells, ultimately attenuating tumor angiogenesis (21). The continuous research on ICIs has highlighted the pivotal role of CD8^+^ T cell-secreted IFN-γ in regulating the tumor microenvironment, demonstrating a broader range of effects (22). To date, some researches have demonstrated that IFN-γ can inhibit tumor angiogenesis by downregulating integrin αVβ3, Dll4, Dll1, and vascular endothelial growth factor A (VEGF-A) (23–28).

In this research, we reported that anti-PD-L1 therapy restrains angiogenesis and boosts vascular standardization in LUAD in an IFN-γ signaling-dependent manner. Mechanistically, IFN-γ, an important cytokine, directly inhibits the expression of the *ANGPT2* and *TEK* (the Tie2 gene name) genes via the JAK1/2-STAT1 signal transduction pathway and indirectly restrains the expression of the *ANGPT2* gene through the AKT–FOXO1 signal transduction pathway in human pulmonary microvascular endothelial cells (HPMECs) and human umbilical vein endothelial cells (HUVECs). The migration, proliferation, and tube formation of ECs are subsequently suppressed by IFN-γ. Our research identified an innovative mechanism by which IFN-γ suppresses tumor angiogenesis and facilitates vascular normalization during PD-L1 blockade therapy. More importantly, these findings may establish a new theoretical foundation for integrating antiangiogenic therapy with ICIs in clinical practice and provide innovative perspectives to reconsider the suboptimal effects, guiding the exploration of new combination therapeutic strategies to enhance anticancer efficacy.

## 2 Materials and methods

### 2.1 Animals

C57BL/6 male mice, aged 6 to 8 weeks, were provided by GemPharmatech Co., Ltd. (Nanjing, China). Mice received a subcutaneous injection of 1×10^6^ Lewis lung cancer (LLC) cells. Mice were randomly allocated into three groups and administered the following treatments: negative control, PD-L1 blockade antibody (on days 0, 3, and 6), or a combination of PD-L1 blockade antibody and ruxolitinib (a JAK1/2 inhibitor) (on days 1 to 5) (n = 5 mice per group). The dosage of the drugs was identical to that described in the previous research (29). The formula for calculating tumor volume is as follows: 0.5 × length × width^2^.

### 2.2 Cell culture

The cell lines HCC827 (KCLB, Cat# 70827, RRID: CVCL_2063) and A549 (ATCC, Cat# CRL-7900, RRID: CVCL_0023) were sourced from Cobioer (Nanjing, China). HCC827 cells were propagated in RPMI 1640 medium (HyClone, Omaha, NE, USA) supplemented with 10% fetal bovine serum (FBS) (Gibco, Grand Island, NY, USA) and 1% penicillin/streptomycin (P/S) (HyClone, Logan, UT, USA). A549 cells were cultured in F12K medium (Boster, Wuhan, China) alongside 10% FBS, 1% P/S. The LLC cells (ATCC Cat# CRL-1642, RRID: CVCL_4358) were propagated in DMEM (HyClone) alongside 10% FBS. Primary HUVECs were purchased from Meisen (Hangzhou, China). HUVECs and HPMECs (Science Cell, Cat# 3000, USA) were grown in endothelial cell medium (ECM) (Science Cell, Cat# 1001, USA) mixed with 10% FBS (Science Cell), 1% ECGS (Science Cell), and 1% P/S (Science Cell). Cells were cultured in a humidified atmosphere with 5% CO_2_ at 37°C.

### 2.3 Cell viability assay

CCK-8 assay kit (Abbkine, Wuhan, China) was used to evaluate cell viability. As previously mentioned (29), cells were plated in 96-well plates at a density of 3,000 cells per well and grown for 12 hours. After the various treatments, a microplate reader (Tecan, USA) was used to determine the absorbance at 450 nm.

### 2.4 Tube formation assay

Primary HUVECs were cultured with ECM for 1 day and subsequently analyzed in a tube formation assay. Matrigel (Corning, Cat# 356237, USA) was allowed to thaw overnight at 4°C. Subsequently, each well of the 96-well plate received 50 μL of Matrigel solution, which was then incubated at 37°C for 60 minutes to promote polymerization. Following that, 2 × 10^4^ HUVECs suspended in ECM were distributed into 96-well plates and then photographed and recorded under a microscope after an incubation period of at least 4 hours.

### 2.5 Transwell assay

ECs were resuspended in ECM without serum and then plated into the upper chamber (1 × 10^5^ cells/100 µL/well). The migration ability of cells was in accordance with the previous protocol (30).

### 2.6 Western blot

According to a previously reported method (29), Western blot analyses were conducted. After cells were lysed by RIPA solution (Beyotime, Shanghai, China), the protein samples were quantified and separated based on their molecular weight via SDS-PAGE gel electrophoresis. Following membrane transfer, the target proteins were visualized by an antigen-antibody reaction and using a chemiluminescence detection system (Tanon, Shanghai, China). **Supplementary Table 2** provides a list of all the antibodies.

### 2.7 Immunofluorescence

In line with a previous protocol (29), immunocytochemistry was performed. Multiplex immunofluorescence (mIF) was conducted utilizing a tyramide signal amplification kit (Servicebio, Cat# G1226, Wuhan, China) following the manufacturer’s guidelines. **Supplementary Table 2** provides a list of all the primary antibodies.

### 2.8 Immunohistochemical staining and quantification

The IHC staining was in accordance with the previous protocol (29). The DAB technique (Genetech, Inc., Cat# GK600705, Shanghai, China) was employed to visualize immunoreactivity. The antibodies utilized are provided in **Supplementary Table 2**. Microvessel density (MVD) was calculated using a modified version of Weidner’s method (31). Each 200× section was examined under 400× magnification, and three distinct regions with the densest microvascular presence were chosen to measure the number of blood vessels.

### 2.9 Real-time quantitative PCR

As our previous protocol (29), RNA isolation and RT-qPCR were conducted. **Supplementary Table 3** lists the primer sequences. The 2^-ΔΔCt^ method was used to assess the relative expression levels, which were normalized to Actin levels.

### 2.10 siRNA transfection

siRNAs designed to target STAT1 were bought from RiboBio (Guangzhou, China). In accordance with the manufacturer’s descriptions, Lipofectamine RNAiMAX (Invitrogen) and siRNAs (50 nmol/L) were gently combined in a serum-free ECM. The transfection solution was introduced into a culture dish, followed by the addition of cell suspensions, which were then incubated for 48 hours. The sequence of the siRNA used for STAT1 was GGAGGAATTGGAACAGAAA.

### 2.11 Chromatin immunoprecipitation

In line with the manufacturer’s descriptions, the Ch-IP assay was conducted by an enzymatic Ch-IP Kit (Cell Signaling Technology, Cat# 9003). The putative binding sites of the STAT1 motif in the *TEK* and *ANGPT2* promoters were predicted via the JASPAR database. **Supplementary Tables 2** and **3** offer comprehensive details about the primers and antibodies utilized. The fragments of the human *TEK* and *ANGPT2* promoters in the immunoprecipitates were identified via RT-qPCR.

### 2.12 Statistical analysis

GraphPad Prism 8.0 performs all statistical analyses. In order to compare the *p* values between the two groups, a two-tailed independent t-test was conducted. In order to compare multiple groups, one-way or two-way ANOVA was employed. Data in the bar charts are displayed as means ± SDs. All *p* values are reported verbatim. *p* < 0.05 is regarded as statistically significant.

## 3 Results

### 3.1 Anti-PD-L1 therapy normalizes tumor vasculature by decreasing the expression of Tie2 and ANGPT2 in a JAK1/2-dependent manner

**Figure 1:**
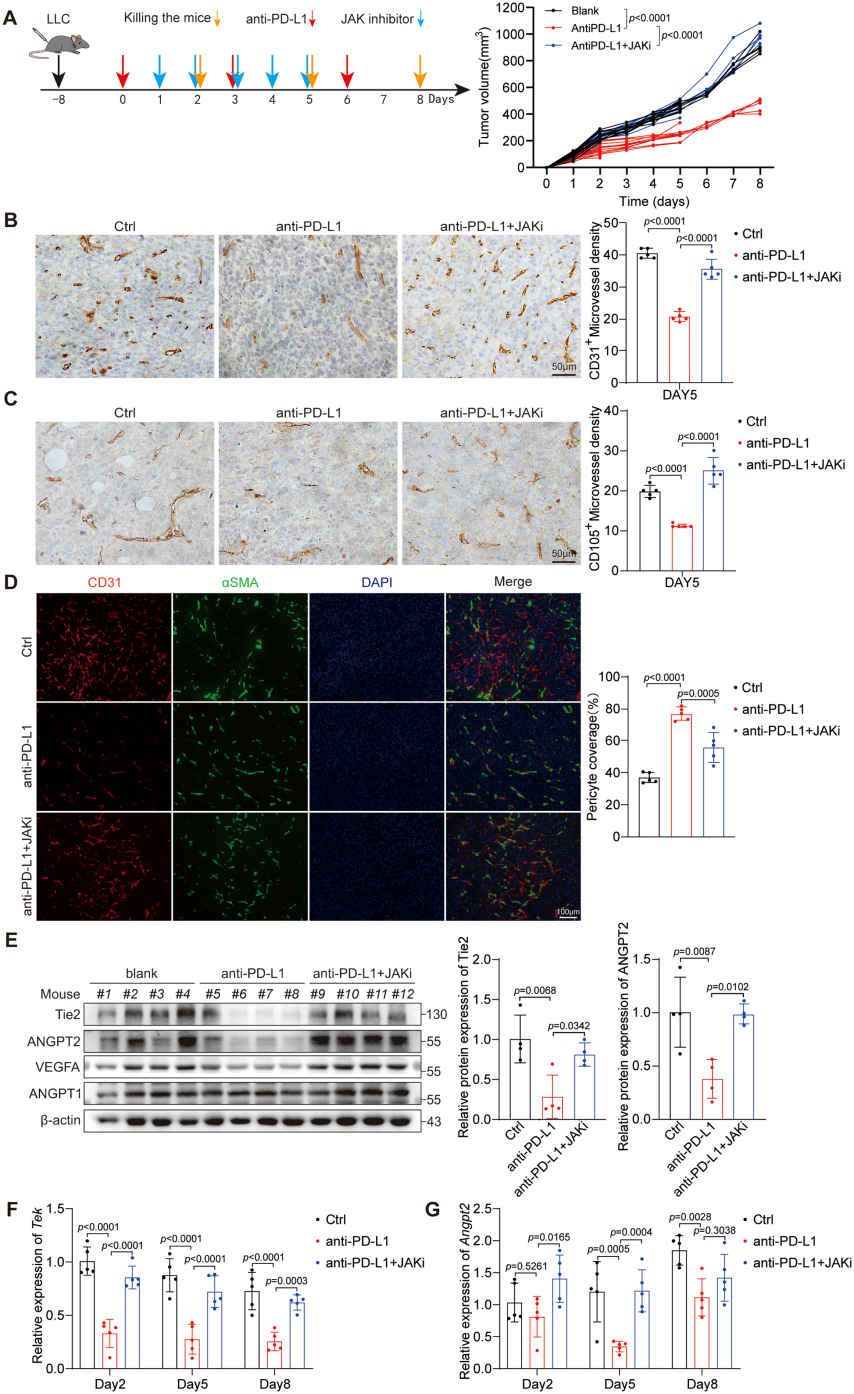
Anti-PD-L1 therapy normalizes tumor vasculature by decreasing the expression of Tie2 and ANGPT2 in a JAK1/2-dependent manner. **(A)** Diagrammatic representation of the research design used to explore the influences of anti-PD-L1 therapy on tumor blood vessels. The red arrows indicate PD-L1 blockade therapy. The blue arrows indicate a cotreatment with anti-PD-L1 antibody in combination with ruxolitinib (JAKi, a JAK1/2 inhibitor). The orange arrows indicate the time point of sacrifice. The volumes of the three different groups of tumors are presented. This *p* values were analyzed via two-way ANOVA. **(B, C)** Representative images showing CD31^+^ vessels **(B)** and CD105^+^ vessels **(C)** in LLC tumor tissues on day 5 as described in **(A)**. The CD31^+^ or CD105^+^ MVD was analyzed separately by one-way ANOVA. Scale bar: 100 μm. **(D)** LLC tumor tissues on day 5 were stained for CD31 (red) and αSMA (green) to determine the percentage of pericyte coverage. Scale bar: 100 μm. The *p* values were evaluated by one-way ANOVA. **(E)** LLC tumor tissue lysates on day 5 were subjected to immunoblotting to assess the expression of specific proteins associated with angiogenesis. And the gray-scale value comparison of the relative expression levels of Tie2 and ANGPT2 proteins is attached. **(F, G)** mRNA from LLC tumor tissues was examined for the expression levels of *Tek* **(F)** and *Angpt2* **(G)** via RT-qPCR. The *p* values were analyzed via one-way ANOVA.

Owing to the rich vascular structure of lung tissue, the progression of lung cancer is intricately linked with tumor neovascularization. To examine the impact of anti-PD-L1 treatment on the vascularization in LUAD, we established the LLC mouse model. Given the essential role of the JAK1/2 pathway in the efficacy of PD-L1 blockade therapy, we conducted further investigations to explore its potential influence on how PD-L1 blockade therapy affects the tumor vascular structures. The experimental mice were selected into different groups: the control group, the anti-PD-L1 antibody group, and the combination group with anti-PD-L1 antibody and ruxolitinib (a JAK1/2 inhibitor). Then, changes in tumor volume were monitored (**Figure 1A**). First, we performed an IHC assay to evaluate the MVD of CD31^+^ cells in mouse tumor tissues on day 5 (**Figure 1B**). Our results indicate that anti-PD-L1 treatment has the potential to decrease the CD31^+^ MVD, whereas ruxolitinib can reverse this effect. Given that CD105 is a reliable marker for actively proliferating ECs (32), we observed the CD105^+^ MVD in tumors and consistently found that anti-PD-L1 treatment reduced the CD105^+^ MVD on day 5 (**Figure 1C**). Similarly, ruxolitinib was able to reverse this inhibitory effect. Moreover, we noted alterations in the MVD of CD31^+^ and CD105^+^ cells, consistent with our findings on days 2 and 8 (**Supplementary Figures S1A-D**). In addition, we performed mIF for CD31 and αSMA to evaluate pericyte coverage of tumor vasculature on day 5 (**Figure 1D**). Our results show that PD-L1 blockade therapy accelerates tumor vascular maturation in LUAD by reducing the MVD of proliferating vessels, whereas ruxolitinib can reverse this effect.

Given the pivotal roles of the VEGF-A/VEGFR2 and ANGPT-Tie2 signaling pathways in regulating vascular development and maturation, we performed an extensive analysis of the protein expression levels of pertinent molecules in murine tumors (**Figure 1E**). Our results demonstrate that PD-L1 blockade treatment effectively suppresses the expression of Tie2, ANGPT2, and VEGF-A through a JAK1/2-dependent mechanism but has no effect on the protein expression of ANGPT1. Notably, compared with no treatment, anti-PD-L1 therapy increased IFN-γ expression and the phosphorylation of STAT1 in tumors. The addition of a JAK1/2 inhibitor abrogated the phosphorylation of STAT1 in anti-PD-L1-treated tumors (**Supplementary Figures S1E, F**). Earlier research has established the inhibitory influence of IFN-γ on VEGF-A expression (25), thus prompting our investigation into the potential of PD-L1 blockade therapy to suppress the expression levels of ANGPT2 and Tie2. The experimental findings uncovered that anti-PD-L1 therapy significantly suppressed *Tek* expression on days 2, 5, and 8 (**Figure 1F**) and *Angpt2* expression on day 5 (**Figure 1G**) upon further investigation in the LLC model. Moreover, the JAK1/2 inhibitor ruxolitinib reversed the suppressive effect of anti-PD-L1 therapy on the expression of Tek and ANGPT2. Taken together, these outcomes indicate that PD-L1 blockade treatment normalizes tumor vasculature and inhibits neovascularization by decreasing the expression of Tie2 and ANGPT2 through a JAK1/2-dependent mechanism.

### 3.2 IFN-γ primarily inhibits the expression of Tie2 and ANGPT2 in ECs

**Figure 2:**
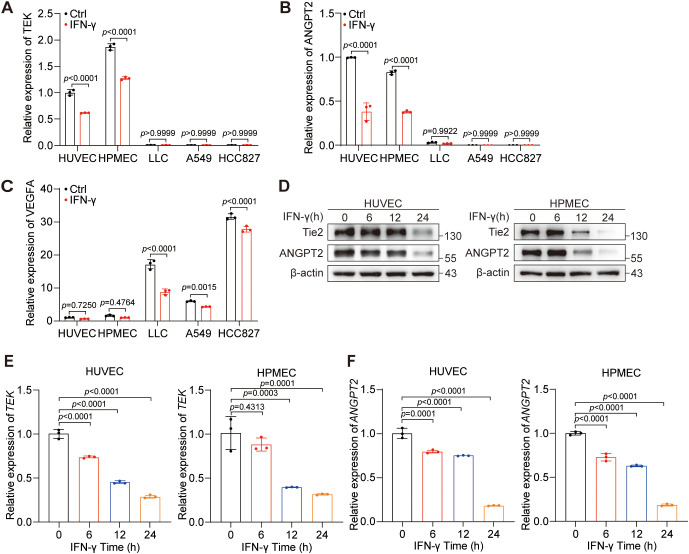
IFN-γ primarily inhibits the expression of Tie2 and ANGPT2 in ECs. **(A–C)** The expression levels of TEK **(A)**, ANGPT2 **(B)**, and VEGF-A **(C)** were evaluated in HUVECs, HPMECs, LLC, HCC827, and A549 cells with or without the treatment of IFN-γ (1000 IU/mL) via RT-qPCR. **(D–F)** HUVECs and HPMECs were stimulated with IFN-γ (1000 IU/mL) for 0, 6, 12, or 24 h. **(D)** Protein expression levels of ANGPT2, total FOXO1, phosphorylated FOXO1 (S256), total AKT, and phosphorylated AKT (S473) were measured using immunoblotting techniques. mRNA levels of *TEK* **(E)** and *ANGPT2* **(F)** were measured via RT–qPCR. The *p* values were analyzed via one-way ANOVA.

Due to the complex cellular elements within the tumor, the expression of ANGPT2, Tie2, and VEGF-A are not limited to a specific type of cell. ANGPT2 and Tie2 are predominantly expressed in ECs (33), whereas VEGF-A is primarily expressed in tumor cells (34). Additionally, IFN-γ is widely acknowledged as the predominant cytokine utilized in anti-PD-L1 therapy (22). Given that tumor cells constitute the majority, we compared their expression in HUVECs, HPMECs, LLCs, A549 cells, and HCC827 cells. Our results uncovered the predominant expression of *ANGPT2* and *TEK* in HUVECs and HPMECs (**Figures 2A, B**), whereas *VEGF-A* was expressed primarily in tumor cells (**Figure 2C**). Notably, IFN-γ was shown to suppress the mRNA expression of *ANGPT2* and *TEK* in HUVECs and HPMECs and to inhibit the mRNA expression of *VEGF-A* in tumor cells. Moreover, we found that the suppression of Tie2 and ANGPT2 protein expression by IFN-γ was most prominent at the 24-hour time point in HUVECs and HPMECs (**Figure 2D**). Intriguingly, the mRNA expression levels of *TEK* and *ANGPT2* were reduced by IFN-γ within approximately 6 hours in both HUVECs and HPMECs (**Figures 2E, F**). In summary, IFN-γ restrains the expression of Tie2 and ANGPT2 in ECs but not in tumor cells.

### 3.3 IFN-γ-mediated activation of STAT1 is required for the reduction in Tie2-ANGPT2 expression

**Figure 3:**
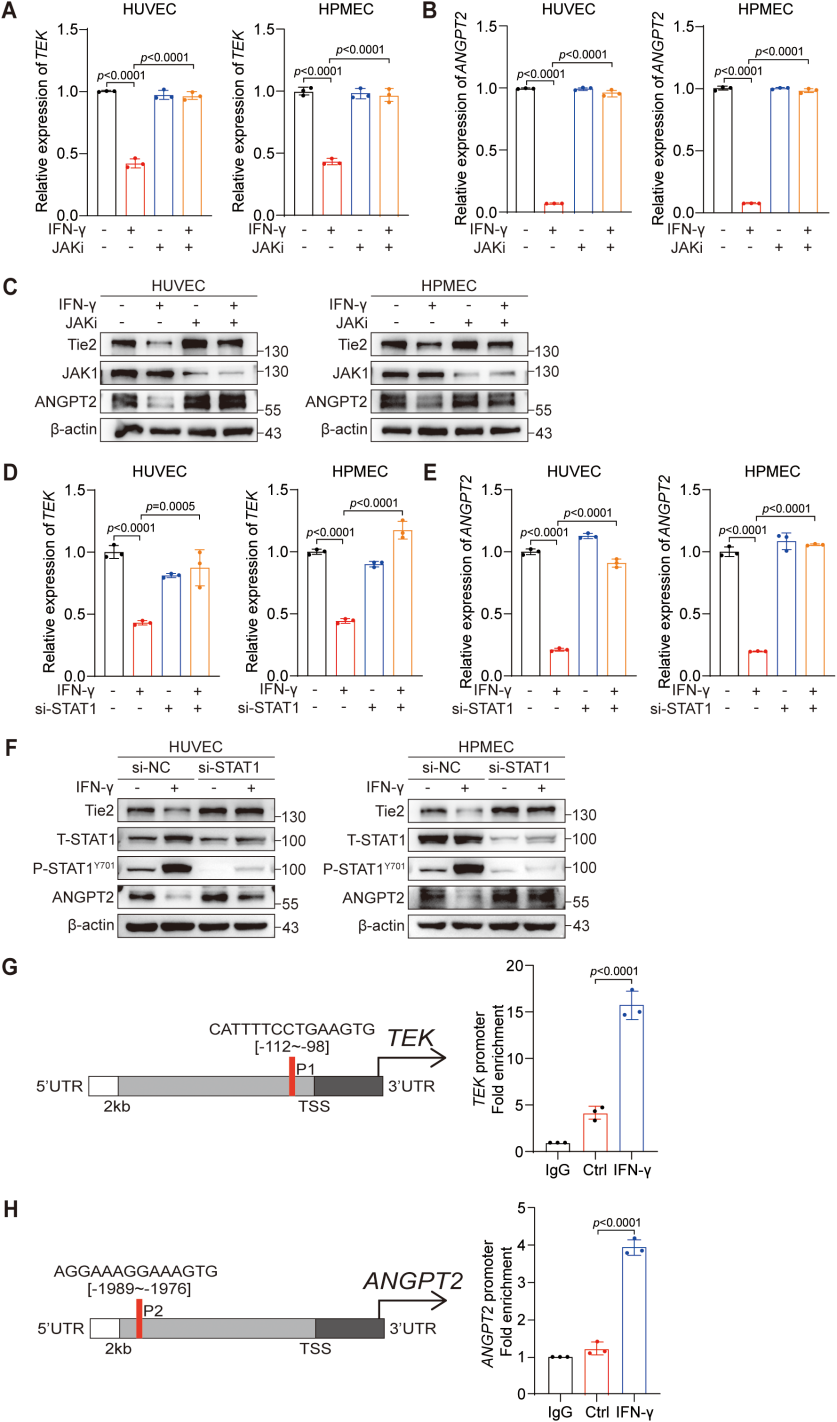
IFN-γ-mediated activation of STAT1 is required for the reduction in Tie2-ANGPT2 expression. **(A–C)** HUVECs and HPMECs were pretreated with a JAKi (ruxolitinib) for 24 h before being stimulated with IFN-γ (1000 IU/mL) for an additional 24 hours. mRNA levels of *TEK* **(A)** and *ANGPT2* **(B)** were assessed by RT-qPCR. **(C)** Protein expression levels of Tie2, ANGPT2, and JAK1 were assessed using immunoblotting techniques. **(D–F)** HUVECs and HPMECs were transfected with siSTAT1 or siNC and subsequently stimulated with IFN-γ for 24 hours. mRNA expression levels of *TEK* **(D)** and *ANGPT2* **(E)** were measured via RT-qPCR. The *p* values were analyzed via one-way ANOVA. **(F)** Protein levels of Tie2, ANGPT2, phosphorylated STAT1 (Y701), and total STAT1 were assessed using immunoblotting techniques. **(G, H)** The putative binding sites of the STAT1 binding motif in the −2 kb *TEK* promoter **(G)** or *ANGPT2* promoter **(H)** were predicted via the JASPAR database. ChIP-qPCR confirmed the binding sites of STAT1 in the promoters of the *TEK* and *ANGPT2* genes.

To further elucidate the specific mechanism through which IFN-γ suppresses ANGPT2 and Tie2, in conjunction with the previous finding that anti-PD-L1 treatment suppresses ANGPT2 and Tie2 via the JAK1/2 signaling pathway, we investigated the role of the JAK1/2 signaling pathway in mediating the inhibition of ANGPT2 and Tie2 by IFN-γ. To this end, we continued to employ ruxolitinib to determine whether IFN-γ regulates ANGPT2 and Tie2 in a JAK1/2-dependent manner. Then, we utilized RT-qPCR and protein immunoblotting to confirm that the suppressive effect of IFN-γ on the protein and mRNA expression levels of ANGPT2 and Tie2 can be counteracted by ruxolitinib (**Figures 3A–C**).

IFN-γ, a key cytokine in response to anti-PD-L1 therapy, primarily exerts its biological effects through the JAK1/2-STAT1 signaling pathway (35). Whether STAT1 plays a pivotal role in the regulatory effect of IFN-γ on ANGPT2 and Tie2 in ECs should be further investigated. We subsequently investigated the involvement of STAT1 in the regulation of ANGPT2 and Tie2 by IFN-γ. Similarly, siRNA was used to silence STAT1 in ECs, confirming the indispensable role of STAT1 in mediating the suppressive effect of IFN-γ on ANGPT2 and Tie2 (**Figures 3D–F**). Collectively, our findings indicate that IFN-γ suppresses the expression of ANGPT2 and Tie2 via the JAK1/2-STAT1 pathway.

Previous research has revealed the role of STAT1 as a repressive transcription factor that can directly bind to the promoter region of *SOX9* and subsequently inhibit its gene expression (36). STAT1 is also considered a negative transcription factor that suppresses tumor angiogenesis (37). These findings prompted us to explore the relationships between STAT1 and the *ANGPT2* and *TEK* genes. Therefore, we utilized the JASPAR database to predict binding sites of STAT1 within the promoter areas of the *TEK* (**Figure 3G**) and *ANGPT2* (**Figure 3H**) genes, selecting those with the highest predicted probability for experimental validation. The putative STAT1 binding sites were validated via ChIP-qPCR assays in the promoter regions of the *TEK* and *ANGPT2* genes. In summary, our results confirm that IFN-γ activates the JAK1/2-STAT1 signaling pathway, leading to the direct interaction of STAT1 with specific sites in the promoter regions of the *ANGPT2* and *TEK* genes.

### 3.4 IFN-γ-mediated activation of the AKT–FOXO1 signaling pathway contributes to the regulation of ANGPT2 but not Tie2 expression

Previous research has demonstrated positive regulation of *ANGPT2* mRNA expression by the transcription factor FOXO1 (38). An increase in nuclear FOXO1 leads to a corresponding increase in *ANGPT2* mRNA expression. The upstream AKT signaling pathway regulates FOXO1 by facilitating its phosphorylation and consequent relocation from the nucleus upon pathway activation. Furthermore, our group revealed that IFN-γ is capable of motivating the PI3K-AKT signaling pathway in LUAD cells (39). Therefore, our objective was to investigate whether the PI3K-AKT signal transduction pathway participates in regulating the suppressive effect of IFN-γ on ANGPT2 expression.

**Figure 4:**
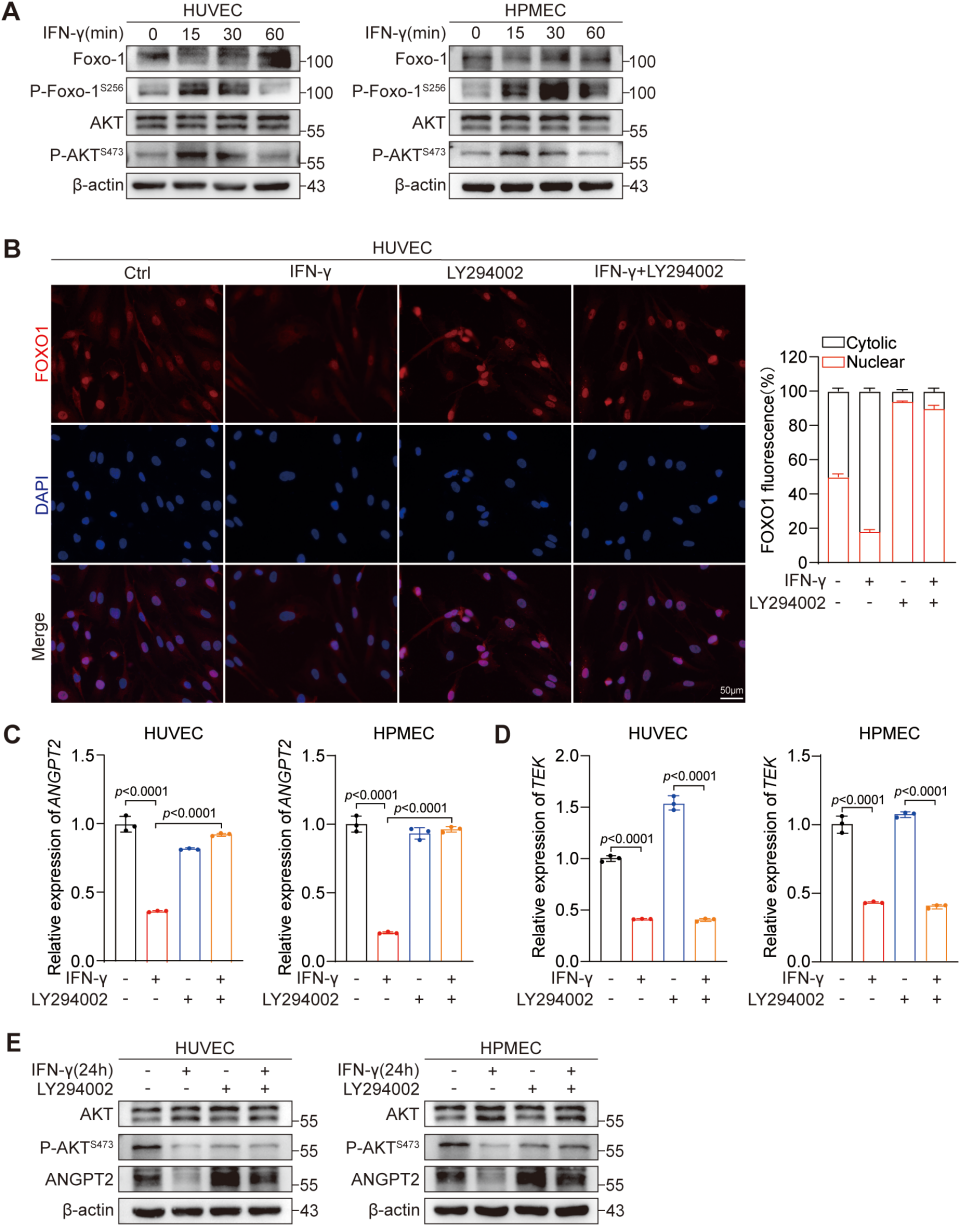
IFN-γ-mediated activation of the AKT–FOXO1 signaling pathway contributes to the regulation of ANGPT2 but not Tie2 expression. **(A)** HUVECs and HPMECs were stimulated with IFN-γ (1000 IU/mL) for 0, 15, 30, or 60 minutes. Protein levels as shown in **Figure 2D** were measured by immunoblotting. **(B)** HUVECs were stimulated with IFN-γ for 24 h or with LY294002 (a PI3K inhibitor) alone or in combination. HUVECs were dual-stained with DAPI (nucleus, blue) and FOXO1 (red). Scale bar: 50 μm. The right chart presents the quantified results of fluorescence digital imaging analysis, showing the percentage expression levels of FOXO1 in both the cytosol and the nucleus. (n = 3). **(C–E)** HUVECs and HPMECs were processed as detailed in **(B)**. mRNA expression levels of *ANGPT2* **(C)** and *TEK* **(D)** were assessed by RT-qPCR. The *p* values were analyzed by one-way ANOVA. **(E)** The specified proteins were assessed using immunoblotting.

To examine the influence of IFN-γ on the PI3K-AKT signaling pathway in ECs, we established a short-term group (within 1 hour) for IFN-γ stimulation. Our findings indicate that IFN-γ increases AKT and FOXO1 phosphorylation at the 1-hour time point (**Figure 4A**). Additionally, the IFN-γ-mediated suppression of ANGPT2 protein expression gradually intensified with prolonged exposure to IFN-γ over the 24-hour period (**Figure 2D**). Similarly, within this same timeframe, the inhibitory effect of IFN-γ on ANGPT2 mRNA expression gradually increased in ECs (**Figures 2E, F**). We hypothesized that following stimulation by IFN-γ, AKT phosphorylation is initially activated, leading to FOXO1 phosphorylation and its nuclear exclusion, which contributes to the downregulation of ANGPT2 expression in ECs. To examine this hypothesis, we employed a PI3K inhibitor LY294002, which effectively suppresses AKT phosphorylation in cells. We subsequently observed the IFN-γ-mediated inhibition of FOXO1 in HUVECs by immunocytochemistry (**Figure 4B**). Our findings revealed that IFN-γ decreased the proportion of cells with nuclear FOXO1 localization, and this effect was reversed when IFN-γ was combined with LY294002. Under the same conditions, LY294002 reversed the suppressive effect of IFN-γ on ANGPT2 at both the protein (**Figure 4E**) and mRNA levels at 24 hours (**Figure 4C**). Notably, the AKT–FOXO1 signaling pathway does not have an impact on the modulation of Tie2 by IFN-γ (**Figure 4D**). In summary, we identified an additional regulatory mechanism through which IFN-γ restrains the expression of ANGPT2 via the AKT-FOXO1 signaling pathway.

### 3.5 IFN-γ inhibits the angiogenic properties of HUVECs

Angiogenesis is often characterized by the intricate interplay of various cell types (4). The biological functions of ECs, the main components of the vascular structure, merit particular attention during angiogenesis. The angiogenic properties of ECs mainly include migration, proliferation, and tube formation. Consequently, we investigated the biological effects of IFN-γ on the migration, proliferation, and tube formation of ECs.

**Figure 5:**
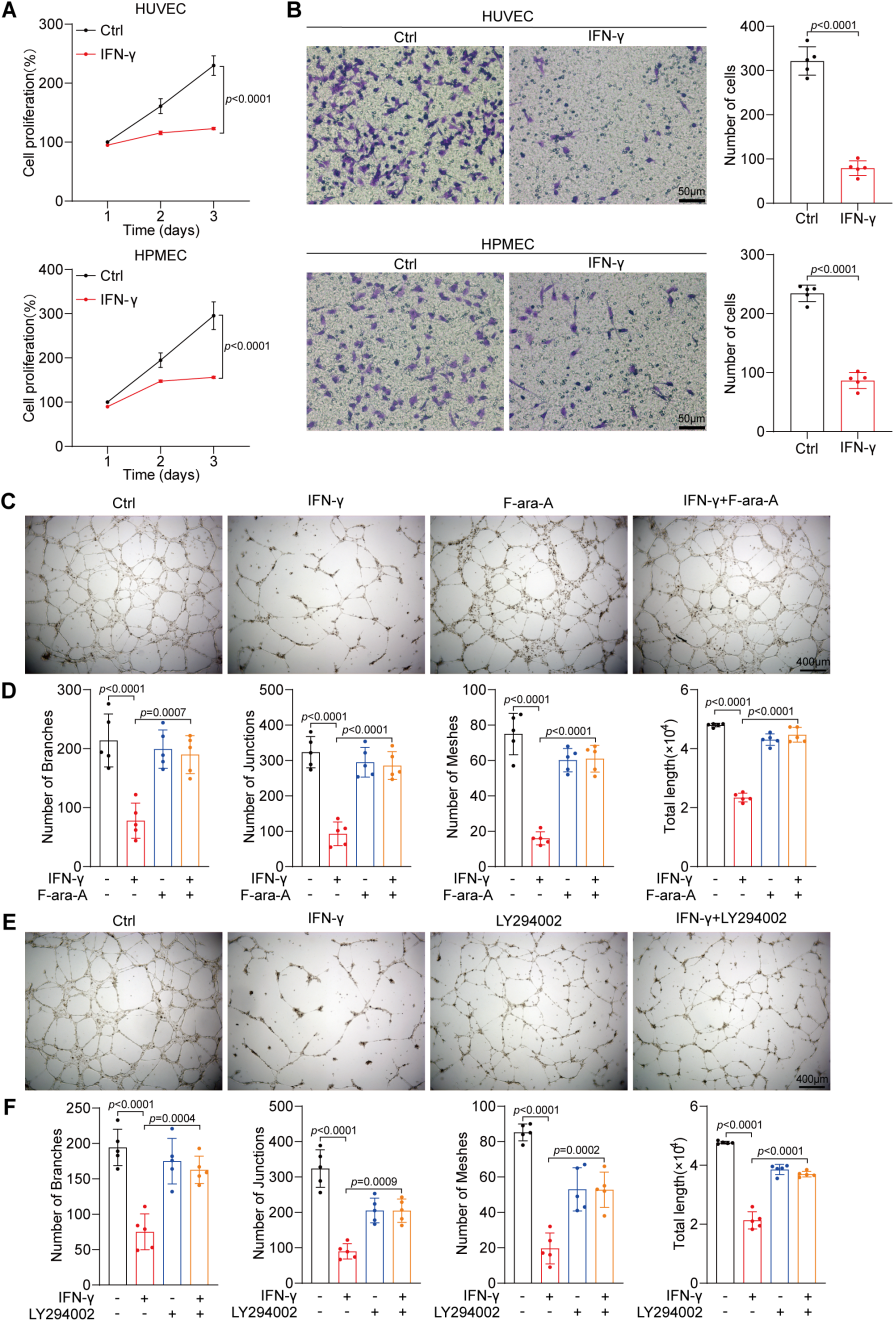
IFN-γ inhibits the angiogenic properties of HUVECs. **(A)** The proliferation of HUVECs and HPMECs treated with IFN-γ (1000 IU/mL) was assessed employing a CCK-8 assay at 24, 48, and 72 hours post-culture. **(B)** Migration assays were conducted on HUVECs and HPMECs after stimulation with IFN-γ (1000 IU/mL) for 24 h. Scale bar: 50 μm. **(C–F)** Tube formation assays of HUVECs and HPMECs. Primary HUVECs were seeded in ECM and matrixgel. Photographs taken 10 h post-seeding were analyzed. HUVECs exhibited a tubular morphology after treatment with IFN-γ (1000 IU/mL) or F-ara-A (fludarabine, a STAT1 inhibitor) alone **(C)** or LY294002 alone **(E)** or in combination. Scale bar: 400 μm. **(D, F)** Quantification of tube formation, encompassing total length, number of branches, number of junctions, and number of meshes, is presented. The *p* values were determined via one-way ANOVA.

Herein, we validated the suppressive influence of IFN-γ on endothelial proliferation through cell viability assays (**Figure 5A**). Next, we conducted Transwell assays to assess that IFN-γ suppresses the migratory capacity of HUVECs and HPMECs (**Figure 5B**). Furthermore, IFN-γ inhibited tube formation in primary HUVECs, reducing the total length, number of branches, number of junctions, and number of meshes. Notably, both a STAT1 inhibitor (F-ara-A, fludarabine) (**Figures 5C, D**) and LY294002 (**Figures 5E, F**) reversed the IFN-γ-induced suppression of HUVEC tube formation.

**Figure 6:**
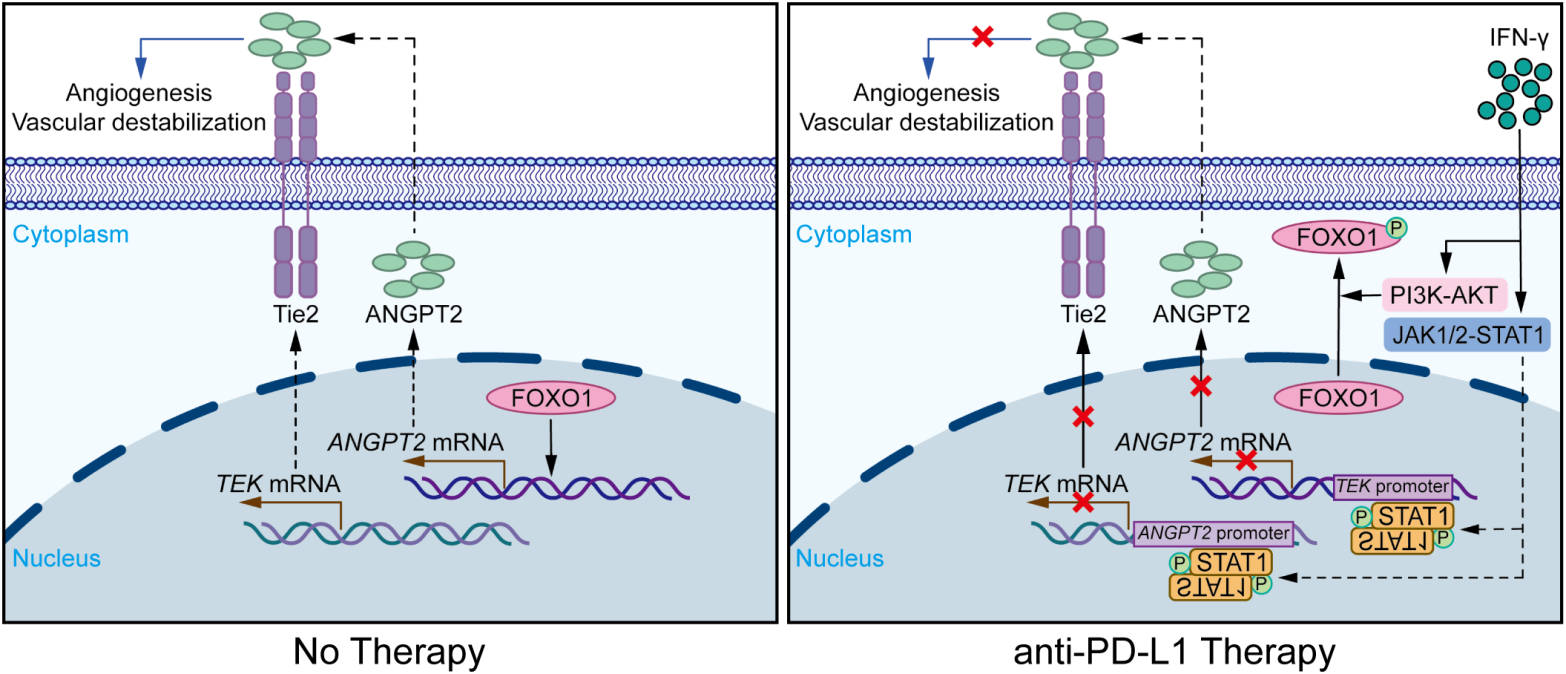
IFN-γ-mediated suppression of ANGPT2-Tie2 in endothelial cells facilitates tumor vascular normalization during immunotherapy. Diagrammatic representation of the mechanism by which PD-L1 blockade therapy modulates IFN-γ to influence the tumor vasculature in ECs through the ANGPT2-Tie2 signaling pathway. IFN-γ motivates the JAK1/2-STAT1 signaling pathway, thereby inhibiting the expression of ANGPT2 and Tie2. Following this process, phosphorylated STAT1 binds to the promoter regions of the *TEK* and *ANGPT2* genes, thereby suppressing their gene expression. Furthermore, IFN-γ phosphorylates FOXO1 through activating the PI3K-AKT pathway, thereby reducing the nuclear activation of FOXO1 on the ANGPT2 gene. Consequently, the synthesis of the Tie2 and ANGPT2 proteins is diminished, contributing to the suppression of the ANGPT2-Tie2 signaling pathway. This effect subsequently suppresses the migration, proliferation, and tube formation of ECs, ultimately contributing to the suppression of tumor angiogenesis and vascular destabilization.

In summary, the findings of this research suggest that IFN-γ stimulates the JAK1/2-STAT1 pathway in ECs during anti-PD-L1 therapy. Accordingly, phosphorylated STAT1 directly binds to the promoter regions of the *ANGPT2* and *TEK* genes, contributing to the inhibition of mRNA transcription and protein synthesis. Additionally, IFN-γ can activate the AKT-FOXO1 signaling pathway in ECs, thereby indirectly suppressing the mRNA expression of *ANGPT2*. Finally, PD-L1 blockade therapy effectively restrains tumor angiogenesis and vascular destabilization in LUAD (**Figure 6**).

## 4 Discussion

This study represents the first demonstration that PD-L1 blockade therapy inhibits tumor angiogenesis and normalizes tumor vessels via IFN-γ-mediated suppression of ANGPT2 and Tie2. We illustrated that anti-PD-L1 treatment can suppress the expression of Tie2, ANGPT2, and VEGF-A, contributing to a reduction in the MVD and an increase in pericyte coverage in LLC model mice. Furthermore, we compared the expression levels of ANGPT2, Tie2, and VEGF-A in various ECs and LUAD cells, demonstrating higher expression of ANGPT2 and Tie2 in ECs compared to LUAD cells. Given that IFN-γ is a widely distributed cytokine produced in response to anti-PD-L1 therapy (22), we validated its capability to inhibit the expression of ANGPT2 and Tie2 in ECs and reduce the expression of VEGF-A in tumor cells. Based on prior research reporting IFN-γ regulation of VEGF-A (25, 40), we concentrated on examining the impact of IFN-γ regulation on ANGPT2 and Tie2. To further investigate and confirm our findings *in vitro*, we found that the expression of ANGPT2 and Tie2 was modulated by the IFN-γ-activated JAK1/2-STAT1 pathway. Given previous reports that STAT1 can negatively modulate gene expression (36), we aimed to confirm that STAT1 exerts negative regulation by binding to the promoter regions of the *ANGPT2* and *TEK* genes. On the basis of the role of FOXO1 in promoting the expression of ANGPT2 in ECs and our previous study demonstrating that IFN-γ induces AKT protein phosphorylation in LUAD cells (38, 39), we showed that IFN-γ facilitates the nuclear export of FOXO1 by promoting AKT protein phosphorylation in ECs, thereby suppressing the positive regulatory effect of FOXO1 on the *ANGPT2* gene. Finally, we also directly observed the suppressive effect of IFN-γ on the biological functions of proliferation and migration in ECs. Additionally, both PI3K inhibitors and STAT1 inhibitors reversed the inhibitory impacts of IFN-γ on HUVEC tube formation.

The VEGF-A/VEGFR2 signaling pathway primarily regulates tumor angiogenesis (34). Previous studies have shown that IFN-γ inhibits VEGF-A expression in various cell types, including macrophages and tumor cells, through both indirect and direct mechanisms (4, 18, 23, 25, 41). VEGF-A is one of the primary target genes of HIF-1α (42). In human glioma cells, STAT1 can abolish HIF-1a activity, thereby reducing VEGF-A expression (43). In IFN-γ-activated monocytic cells, VEGF-A is suppressed through a post-transcriptional pathway. Ray and colleagues reported that although IFN-γ induced persistent VEGF-A mRNA expression, translation was suppressed by the delayed binding of the IFN-γ-activated inhibitor of translation (GAIT) complex to a specific element in the 3’UTR. This binding leads to translational silencing and decreased VEGF-A synthesis (44). Our study revealed that anti-PD-L1 therapy reduced VEGF-A expression in LLC tumors in a JAK1/2-dependent manner. Consistent with previous reports, we showed that IFN-γ inhibits VEGF-A expression in LLC, A549, and HCC827 cell lines *in vitro*. In addition to promoting angiogenesis, VEGF-A plays a critical role in regulating tumor vasculature normalization (45). By suppressing VEGF-A expression, the abnormal proliferation and leakiness of immature blood vessels in tumor tissues can be mitigated, ultimately promoting vascular normalization (46, 47). In brief, those findings further elucidate the molecular mechanisms underpinning the integration of anti-angiogenic therapies with ICIs, such as atezolizumab and bevacizumab.

The ANGPT-Tie2 signaling pathway primarily modulates tumor vascular normalization (6). Notably, an increase in Tie2 phosphorylation can facilitate tumor vessel normalization (48). In contrast, this investigation revealed that inhibiting Tie2 promotes tumor vascular normalization. Additionally, the inhibition of ANGPT2 further restrains the malignant progression of tumor blood vessels. However, PD-L1 blockade treatment exerted a negligible influence on the expression of ANGPT1. Furthermore, although STAT1 acts as a positive regulatory transcription factor involved in various biological activities, it also functions as a negative transcription factor. In this study, STAT1 exerts negative regulation by binding to the promoter regions of the *ANGPT2* and *TEK* genes. This finding provides further evidence for the bidirectional regulatory role of STAT1 as a transcription factor.

IFN-γ is not the sole efficacious antitumor cytokine generated by ICIs. TNF-α also functions as a crucial antitumor factor (49–51). IFN-γ induces tumor vascular regression, while TNF-α bursts them (23). The drawback is that this article did not to investigate how anti-PD-L1 therapy regulates tumor blood vessels via TNF-α. Indeed, the mechanisms of action of IFN-γ on tumor blood vessels are multifaceted and intricate (4, 40). IFN-γ can hinder tumor angiogenesis through targeting the Notch-Dll4/Dll1 pathways or integrins (24, 26–28, 52, 53). More critically, this paper, for the initial time, elaborates that IFN-γ modulates the expression of ANGPT2 and Tie2 via the AKT-FOXO1 and JAK1/2-STAT1 pathways, thereby providing direct evidence for the normalization of tumor vascular structures regulated by IFN-γ in immunotherapy.

The findings of this research, in conjunction with those from prior studies, demonstrate that immunotherapy exerts an inhibitory effect on tumor angiogenesis and normalizes the tumor vascular system (18–20). By elucidating novel mechanisms, we have provided additional evidence for the clinical integration of immunotherapy with antiangiogenic therapy, thereby prompting further exploration of optimized combination treatment strategies. First, based on the suppression of ANGPT2, Tie2, and VEGF-A expression by anti-PD-L1 treatment, we can enhance antitumor effects by combining targeted therapies against ANGPT2, Tie2, and VEGF-A with anti-PD-L1 treatment. Second, due to the lack of an effect of PD-L1 blockade treatment on ANGPT1, the upregulation of ANGPT1 in combination with ICIs may enhance antitumor effects by facilitating tumor vascular normalization. However, the implementation, therapeutic efficacy, and adverse reactions of these strategies still require further exploration. Additionally, this study lacked direct validation of its findings in human LUAD tissue samples before and after immunotherapy.

In conclusion, this study innovatively illustrates that IFN-γ-mediated inhibition of ANGPT2-Tie2 in ECs inhibits angiogenesis and normalizes tumor vasculature during immunotherapy. Specifically, IFN-γ exerts a direct dual-inhibitory effect on the expression of ANGPT2 and Tie2 by activating the AKT-FOXO1 and JAK1/2-STAT1 signaling pathways. Our experimental findings reveal a novel mechanism underlying the mutual regulation between the immune system and tumor vasculature within the tumor microenvironment, guiding the design of treatments that integrate ICIs with antiangiogenic therapy in clinical practice.

## Data Availability

The datasets presented in this study can be found in online repositories. The names of the repository/repositories and accession number(s) can be found in the article/**Supplementary Material**.
