# Supplementary material for: IFN-γ-mediated suppression of ANGPT2-Tie2 in endothelial cells facilitates tumor vascular normalization during immunotherapy
Source: Front Immunol. 2025 Apr 30;16:1551322. doi: 10.3389/fimmu.2025.1551322 (PMC12075545; doi:10.3389/fimmu.2025.1551322)
Supplement: Supplementary file 1 [file DataSheet1.docx]

Supplementary Material

**1. Supplementary Figures and Tables**

**1.1 Supplementary Figure 1**


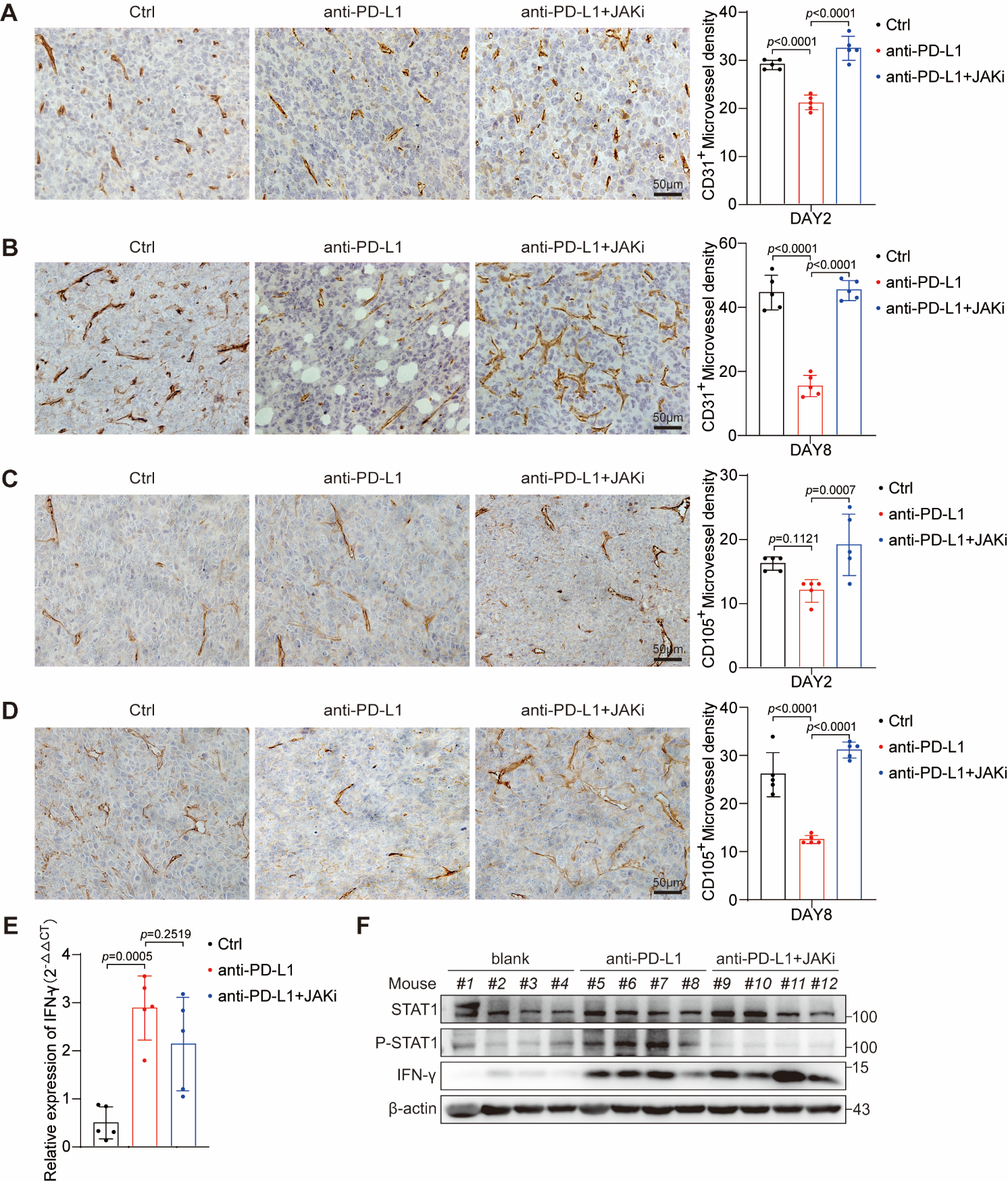


**Supplementary Figure 1** **Anti-PD-L1 therapy suppresses the CD31^+^ and CD105^+^ MVD via the JAK1/2 signaling pathway on days 2 and 8.** (A - D) Representative images showing CD31^+^ vessels (A and B) and CD105^+^ vessels (C and D) in LLC tumor tissues on days 2 and 5. The CD31^+^ or CD105^+^ MVD was analyzed separately via one-way ANOVA. The *p* values were determined via one-way ANOVA. Scale bar: 50 μm. The data shown are the means ± SDs. (E and F) On day 5 after the initiation of anti-PD-L1 therapy, the expression levels of IFN-γ in LLC tumor tissues were examined via RT-qPCR (E), and LLC tumor tissue lysates were subjected to immunoblotting to assess the expression of specific proteins (F). The *p* values were evaluated by one-way ANOVA.

**1.2 Supplementary Tables**

**1.2.1 Supplementary Table 1: Key Reagents Table**

| **Reagents** | **SOURCE** | **IDENTIFIER** |
| --- | --- | --- |
| Human IFN-γ Protein | Novus | Cat# NBP2-34992 |
| Mouse IFN-γ Protein | Novus | Cat# NBP2-35071 |
| Ruxolitinib | Selleck | Cat# S1378 |
| Fludarabine | MCE | Cat# HY-B0069 |
| LY294002 | MCE | Cat# HY-10108 |
| HiScript IV RT SuperMix for qPCR (+gDNA wiper) | Vazyme | Cat# R423-01 |
| ChamQ Blue Universal SYBR qPCR Master Mix | Vazyme | Cat# Q312-02 |
| Enzymatic Chromatin IP Kit | CST | Cat# 9003 |
| TSAPLus Kit | Servicebio | Cat# G1226 |
| RNAiso | Takara | Cat# 9109 |
| Lipofectamine RNAiMAX Transfection Reagent | Invitrogen | Cat# 13778030 |

**1.2.2 Supplementary Table 2: Key Antibodies Table**

| **Antibodies** | | | | |
| --- | --- | --- | --- | --- |
| **Antibodies** | **Application** | **Supplier** | **Catalog Number** | **Dilution** |
| anti-CD31 | IHC | Abcam | Cat# ab182981 | 1:100 |
| anti-CD105 | IHC | Abcam | Cat# ab221675 | 1:100 |
| anti-αSMA | IF | CST | Cat# 19245 | 1:200 |
| anti-CD31 | IF | R&D | Cat# AF3628 | 5 µg/mL |
| anti-Tie2 | WB | R&D | Cat# AF762 | 0.1 µg/mL |
| anti-ANGPT2 | WB | R&D | Cat# AF7186 | 0.1 µg/mL |
| anti-ANGPT1 | WB | Abcam | Cat# ab183701 | 1:1000 |
| anti-VEGF-A | WB | Abcam | Cat# ab214424 | 1:1000 |
| anti-Tie2 | WB | R&D | Cat# AF313 | 0.1 µg/mL |
| anti-ANGPT2 | WB | R&D | Cat# AF623 | 1 µg/mL |
| anti-JAK1 | WB | CST | Cat# 3344 | 1:1000 |
| anti-T-STAT1 | WB | CST | Cat# 14994 | 1:1000 |
| anti-P-STAT1(Y701) | WB | CST | Cat# 7649 | 1:1000 |
| anti-AKT | WB | ABclonal | Cat# A18675 | 1:2000 |
| anti-P-AKT(S473) | WB | ABclonal | Cat# AP1208 | 1:1000 |
| anti-FOXO1 | WB | CST | Cat# 2880 | 1:1000 |
| anti-P-FOXO1(S256) | WB | CST | Cat# 9461 | 1:1000 |
| anti-IFN-γ | WB | CST | Cat# 98139 | 1:1000 |
| anti-β-actin | WB | Protein-tech | Cat# 66009-1-Ig | 1:10000 |

**1.2.3 Supplementary Table 3: Key Primers Table**

| **Primers (5′–3′)** | | |
| --- | --- | --- |
| **Gene** | **Forward** | **Reverse** |
| *Tek* | ACGGACCATGAAGATGCGTCAACAA | TCACATCTCCGAACAATCAGCCTGG |
| *Angpt2* | ACCGGTCAGCACCGCTACGTG | TGCGTCAAACCACCAGCCTCCTG |
| *IFN-γ* | GCCACGGCACAGTCATTGA | TGCTGATGGCCTGATTGTCTT |
| *Actin* | CATTGCTGACAGGATGCAGAAGG | TGCTGGAAGGTGGACAGTGAGG |
| *VEGF-A* | AGGGCAGAATCATCACGAAGT | AGGGTCTCGATTGGATGGCA |
| *TEK* | CCAGGATGGCAGGGGCTCCA | GGTAGCGGCCAGCCAGAAGC |
| *ANGPT2* | AACTTTCGGAAGAGCATGGAC | CGAGTCATCGTATTCGAGCGG |
| *ACTIN* | TGACGTGGACATCCGCAAAG | TCTTCATTGTGCTGGGTGCC |
| ChIP P1 | CTGAGCCAGTATTTGAACCC | CTCATCATCGACCTTCATGC |
| ChIP P2 | CTACAGGAAGATAACGGCT | ATCCGAATCAATCACTTTC |
